# Analysis Model of Consumer Sentiment Tendency of Commodities in E-Commerce

**DOI:** 10.3389/fpsyg.2022.887923

**Published:** 2022-06-09

**Authors:** Hui Yao

**Affiliations:** Zhejiang Vocational College of Special Education, Hangzhou, China

**Keywords:** sentiment tendency, commodities, e-commerce, brand loyalty, product review, product quality, promotion

## Abstract

Users are increasingly turning to the internet to acquire and consume goods. Online purchasing builds demand between customers in modern years. E-commerce (e-commerce) is a business strategy that allows individuals and businesses to buy and sell goods and services through the Internet. Ecommerce can be used on computers, tablets, cellphones, and other smart devices, and it operates in four key market categories. The way individuals buy and consume goods and services has changed as a result of e-commerce. People are increasingly using their computers and smart devices to place orders for things that can be delivered quickly to their homes. In the 1960s, ecommerce made use of an electronic system called electronic data interchange to help in document conversion. In the world of e-commerce, Amazon is a monster. It is, in reality, the world's largest online store, and it is still growing. As a result, it has become a significant roadblock in the retail industry, prompting some major merchants to rethink their plans and adjust their focus. This article is based on literary reviews. Developing a research framework for consumer trends, particularly in terms of purchasing behavior, is very much necessary. The sample size for this investigation was determined using a simple rule of thumb for successful partial least squares structural equation modeling (PLS-SEM) estimation. Consumer sentiment tendencies play a major role in this research. This research's most valuable factors include a promotion, price, brand loyalty, product review, and product quality. We looked into how these aspects analyzed a customer's tendency. These are the primary topics of discussion in this study.

## Introduction

After globalization, E-commerce (electronic commerce) plays a vital role in the business world with rapid development and popularization because of advancement and emerging of technologies as well as consumers purchasing power and internet users (Chawla and Kumar, 2021). E-commerce is the electronic buying or selling of goods *via* internet services. Mobile commerce, online fund transfer, logistics, digital marketing, online transaction process, electronic data interchange (EDI), inventory system, and online data collection systems are used in e-commerce. E-commerce, which is the major sector of the electronics industry, is driven by technological developments the semiconductor industry (Kwilinski et al., 2019). Internet behaves as a specific platform that connects buyers and sellers for the exchange of products while using an online website (Khan et al., 2021). E-commerce businesses employ the buying and selling of commodities also in Business to Business (B2B). The electronic business helps to support E-commerce (Babenko et al., 2019). E-commerce can be classified into three divisions, such as electronic retail, online markets, and electronic transactions (Saleem et al., 2019). E-commerce utilizes the internet for a transaction's life cycle, although it also might use other technologies, such as e-mail, social media, and apps. E-commerce transactions include the buying of goods (such as books, electronic products, beauty products, costumes, etc. from Amazon, Flip kart, eBay) and services (such as music or videos downloads in the form of digital distribution, such as iTunes Store or Google play store) (Singhal et al., 2020).

Year after year, China's E-commerce presence among emerging market countries expands. China's internet shopping revenue totaled $253 billion in the first quarter of 2015, taking account for 10% of total Chinese consumer retail sales in that period, with 668 million Internet users (Millward, 2015). China is the world's largest e-commerce market in sales value, with an estimated US$899 billion in 2016 (Rahman et al., 2018). The consumer is the king of the business world. Factors influence the consumer's tendency to buy a commodity in E-commerce based on the income of consumers, information about a product, advertisement, price of the product, product brand, brand loyalty, product quality, product reviews, etc. Therefore, consumer behavior is important in the online purchase of the commodity (Xie and Lou, 2020). The process through which consumers decide to buy things through E-commerce is known as online consumer behavior. The actions themselves, such as identifying the problem of making a purchase, are based on constantly changing expectations and requirements (Vanhala et al., 2020). While each shopper's demands are different, the new expectations that are driving online consumer behavior are anchored in commonality (Dwivedi et al., 2021).

**Figure 1:**
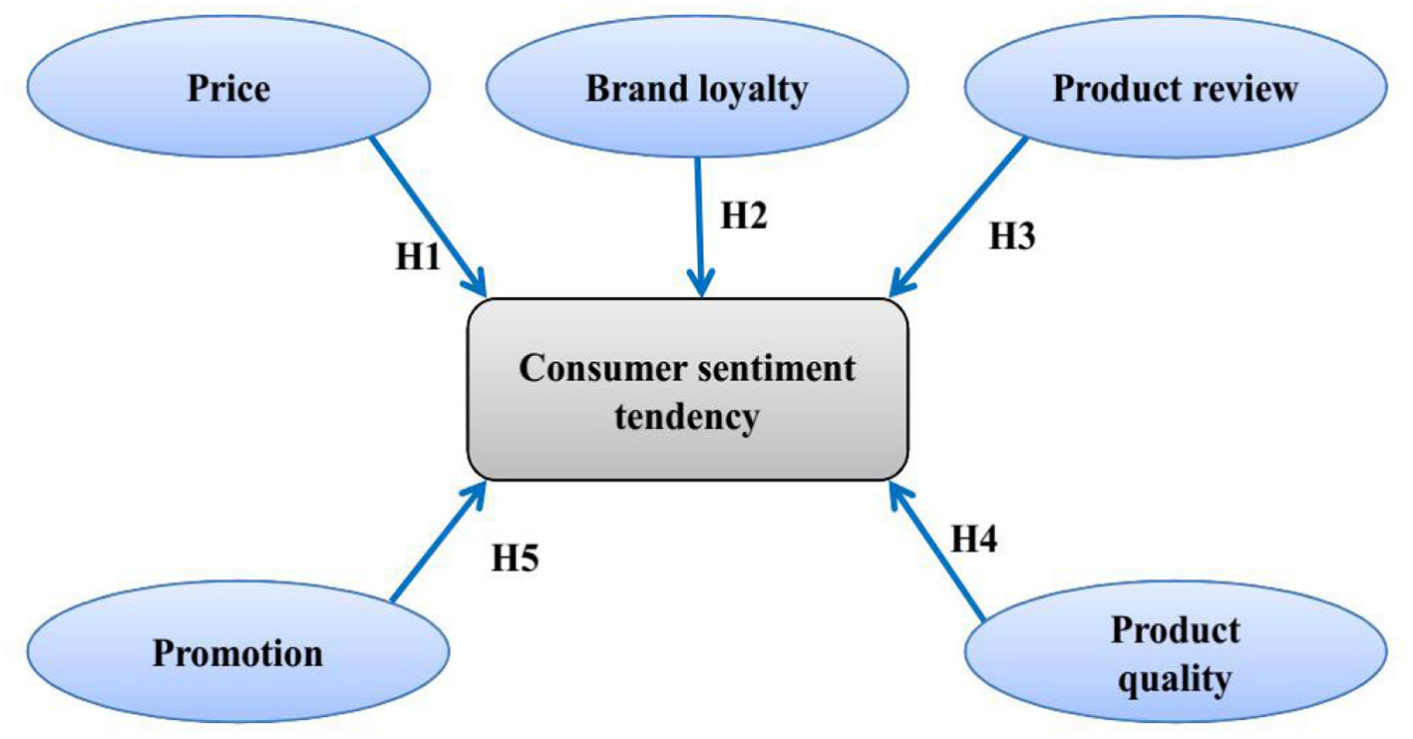
Conceptual framework.

Consumers get benefits from online purchasing commodities because of the virtuality E-commerce platform, but, also, some challenges regarding products available on platforms, such as inconsistencies between specific information and actual goods, poor quality of commodities, unsatisfactory after-sales service, and so on, are some of the drawbacks of internet purchase of commodities (Chen et al., 2018). Analyzing the sentiment tendency of consumer evaluations can aid E-commerce enterprises in improving service quality and customer satisfaction (Yang et al., 2020). Sentiment Analysis for product reviews, otherwise known as text orientation analysis, is the method of evaluating subjective comments text with the emotional color of the customer automatically (Alsaeedi and Khan, 2019). This study analyzes the consumer sentiment tendency of commodities in E-commerce by using these factors: price, brand loyalty, product review, product quality, and promotion (Figure 1).

## Literature Review

Yang et al. (2020) projected a recent sentiment analysis model-SLCABG, which was based on the sentiment usage and linked convolutional neural network (CNN) and attention-based bidirectional gated recurrent unit (BiGRU). According to the data collection, this paper mentioned the actual book rating, trained and tested of the well-known Chinese e-commerce website dangdang.com, which was Chinese based. Experimental results explained that the idea can efficiently maintain the accomplishment of text perception analysis. Tseng et al. (2018) examined the information that influenced the price of products and set up the new idea for access of financial value based on Internet sentiment analysis. Significant news events had a collision on the trading price of electronic goods, and the results exhibited that they may become better the correctness of assessing financial value's approximate calculation. Therefore, the handout of this study was to suggest a fresh predict model for the financial value of e-commerce goods created.

Zhang et al. (2020) divided e-commerce text ideas into three classifiables: keywords, value objects, and emotional themes. It was identified based on a structured emotion dictionary. Specific test steps: First, the tf-idf algorithm for separated keyword study; next, by using the idea of part of speech, linked keywords likeness evaluation object extraction, and situated on the typical feature of parts of speech and activity rules set to separate emotional resources; and, last, created a promptory based on emotional resources, and made an opposite promptory for various rating objects with dissimilar polarities for the same sentimental terms. Experimental results confirmed that the emotional dictionary made into this paper had a positive result on the emotional categorization of e-commerce subject matter examinations.

Yang et al. (2022) questioned: How factors that influenced shopping websites affect online shopping decisions? And what are the key factors touching consumer shopping decision? The most desired criteria impacting consumer decision-making are sales volume and the number of high-quality negative comments; however, the number of comments and the number of comments with photos are very minor factors that have the least impact on shop type and video display products. The proposed approach can be utilized as a standardized evaluation platform for contact research, which is an important component of procurement research (Zhang and Zhong, 2019). In e-commerce systems, the search for and acceptance of feelings and ideas were thought to signify a level of trust among customers while buying. The transcendence aspect is used to compute the trust spread. According to the findings, emotional similarity analysis can be a useful tool for determining user trust in e-commerce systems (Table 1).

**Table 1:** Literature survey.

**Author/year**	**Techniques used**	**Methodology**	**Research findings**
Yang et al. (2020)	Convolutional Neural Network (CNN) and attention-based Bidirectional Gated Recurrent Unit (BiGRU).	In reviews, the Sentiment Lexicon is utilized to enhance emotional characteristics.	This concept could help to keep track of text perception analysis.
Tseng et al. (2018)	Semantic analysis algorithm.	A novel forecasting model for the pricing of e-commerce products has been proposed.	Advised the creation of a new forecast model for the financial value of e-commerce items.
Zhang et al. (2020)	tf-idf algorithm.	A reverse dictionary for the same emotional phrases is constructed for different assessment objects with varied polarity.	The emotional categorization of e-commerce course exams improved as a result of the emotion lexicon produced in this study.
Yang et al. (2022)	Network evolutionand Sales distribution analysis.	The best-simulated sales distribution is quite close to the real thing, and it determines whether the network evolution technology is applicable.	The suggested method may be utilized to provide a standardized evaluation platform for communication research, which is an important part of procurement research.
Zhang and Zhong (2019)	Shortest path algorithm.	A large-scale E-commerce website reviews dataset is gathered to test the algorithms' accuracy and model feasibility.	Emotional similarity analysis, according to the findings, can be a beneficial method for determining user confidence in e-commerce systems.

## Theoretical Framework

### Analysis of Online Users Previous Impulsive Buying Behaviors

When a customer buys some goods and services without any preplanning, then it is called impulsive buying. Such decisions are triggered by feelings and emotions within a second. Because of online shopping platforms, impulsive buying behavior has increased among customers (Aragoncillo and Orus, 2018). Impulsive buying is done by some situational factors such as time, browsing inside the store, the presence of others, and money (Dergipark.gov.tr., n.d.). Online e-commerce and social business research have become increasingly pervasive in purchases, although there is a lack of systematic research into a particular phenomenon in the information system paradigm. Meta-analysis is used to research online impulsive buying (Zhao et al., 2021). The social presence of live streaming sites and live streamers, viewers, and telepresence has a positive and significant influence on consumer confidence and flow status, which stimulates impulsive buying behavior and personal power as moderators (Ming et al., 2021). An existing study shows the relationship between online impulsive buying behavior and hedonic and utilitarian motivation under a Chinese business platform, which consists of 585 valid questionaries. As a result, hedonic and utilitarian motivation positively affects online impulsive buying behavior (Akram et al., 2021).

Data from outboard Chinese travelers are collected to find the relationship between impulsive buying behaviors for cognitive and affective and, also, to find the loyalty/satisfaction in airport management. The result of this existing study shows that the relationship is positive between impulsive buying and traveler satisfaction/loyalty under the condition of airport duty-free shopping (Lee et al., 2021). The pleasure and the shopping pleasure moderate the effect of the atmosphere of the store on impulse purchasing behavior by hedonic shopping motives (Hashmi et al., 2020). A study of Iran's Instagram users shows the consumer's impulsive buying behavior on Instagram by evaluating the influence of experience flow and hedonic browsing on impulsive buying. About 635 Iranian Instagram users have participated in this survey, and the data analysis was managed by structural equations. The result shows a positive effect on impulse buying and the flow of experience (Shahpasandi et al., 2020). Some of the factors that affect online impulsive buying are materialism, online platform quality, sales promotion online, fashion consciousness. During COVID-19, online platforms have increased significantly, and the customers' trend of impulsive buying behavior is also varied (Sritanakorn and Nuangjamnong, 2021).

### Impact of Economic Development Level in Investigating the Consumer's Sentiments

By determination of the consumer feedback on overall health of an economy is based on the consumer's sentiments (Liberto, 2021). Consumer sentiments show people's feelings about the health of the economy in the short term, the prospects for long-term economic growth, and current financial health, and it is considered a successful economic indicator (McArthur and Sachs, 2001; Kwortnik and Ross Jr, 2007; Luo and Huang, 2021). Using 723,363 Chinese-text online reviews to explore the experiences of Chinese economic hotel guests using in-depth learning and fine-grained sentiment analysis (Lagast et al., 2017). The location is associated with the most positive emotions, followed by price, booking experience, facilities, pictures, and service (Diao et al., 2014; Tussyadiah and Zach, 2017). Key features associated with negative sentiments consist of air conditioning, TV sets, elevators, toilet bowls, invoices, sound insulation, beds, WiFi signals, refunds, towels, shoes, windows, hair dryers, and toilets. Negative and positive words to infer users' sentiments are compared (Jiang et al., 2021). A substitute approach and a complete understanding of the experiences and sentiment of guests of the Chinese economic hotel are provided by the research. The evaluation is done to obtain PVAR (panel vector auto-recreation) and dimension-specific perception models, and use feature-level perception analysis to evaluate their effects on product sales using the Movie Panel database. The three-dimensional-specific emotions, such as plot, star, and genre, are positively related to film sales. In terms of consumer sentiment preferences, the positive relation of movie sales to plot sentiment has been compared to the star sentiment for low-budget movies (Szymaniak and Zajenkowski, 2021).

### Affective Stimuli

#### Arousal

Arousal was explained in the stages of emotional agitation, which was represented by values from 1 to 9. The value one represents the high agitation, and nine represents the lower agitation. The pleasant feelings were incorporated with the rewards, which have high arousal values (Szymaniak and Zajenkowski, 2021). Yang et al. (2021) determined how the consumers' perceived values affect their impulse buying behavior in E-commerce, and the environmental impulses were created a high impact on consumer-perceived values. Consumer impulse buying was affected by advertisements with high involvement and low involvement of products. The customers want to get a pleasant feeling from the advertisement through popular celebrities, which enhances the arousal values Parmar et al. (2020).

#### Pleasure

Customer satisfaction was illustrated by how the employees treated the customers and the quality of products. Tong and Shen (2021) explained the customer satisfaction model by using restrained discount promotions and the customer psychological mechanism. These discount promotions and product qualities enhance customer satisfaction. Li et al. (2021) examined the factors affecting their customer satisfaction by using e-banking services. The cloud services, security, e-learning, and service quality were influenced by customer satisfaction while using internet banking services.

#### Positive Emotion

The employees must understand and gather the customers' emotions and ability to provide the exact information about the products, express good behavior, and provide good quality products. Those services created positive emotions. Xiong (2020) introduced customer sentiment and critical factors of impulsive purchase behavior in the e-shopping environment. The customer sentiments were included with the improvement of a payment mode and increased the positive emotions to customers. Fengliang and Jianhong (2021) described the investigation of customer impulse buying intention of direct broadcasting commerce, which has a positive effect. The flow of mechanisms has a positive impact on impulse buying intention.

#### Negative Emotion

The unnecessary services, experiences, qualities, and consumption created negative emotions. Mukhtar et al. (2021) elaborated on how materialism and depression affected their buying intentions. The depression has moderated the confidence in materialistic and impulse buying. Huang and Wang (2021) proposed the data gathered from the opinion poll were examined and treated by using a trial-and-error process. Impulsive consumption has a negative impact on prestige platforms and the recycling process. This provides a new point of view for new technology applications and the effects on customer behavior.

## Hypothesis Development

### Price

The price of a product has a considerable impact on a customer's decision to purchase it. The consumer's perception of price clarifies information about a product and gives it relevance (Ghali-Zinoubi and Toukabri, 2019). Pricing plays an important role in purchasing decisions, especially for commonly purchased things, and it influences the retailer, product, and brand to choose. Consumers are quite intelligent when it comes to selecting what benefits they want to obtain from purchasing things or services (Safitri, 2018). Hence, the price of a product is an important factor that influences consumer tendency in buying commodities in E-commerce.

**H1:** *The price of a product influences consumers' tendency in buying commodities in E-commerce*.

### Brand Loyalty

Brand loyalty is defined as a consumer's desire to regularly purchase a branded product or service of his or her choice regardless of external influences or factors that would drive the consumer to switch brands (Holmes et al., 2020). Many experts have recently stressed in research that “loyalty is more profitable” since the expense of obtaining new consumers far outweighs the cost of retaining existing customers, and loyal customers, more crucially, encourage or recommend others (Agha et al., 2021). Hence, brand loyalty of a product influences consumer tendency in buying commodities in E-commerce.

**H2:** *Brand loyalty of a product influences consumer tendency in buying commodities in E-commerce*.

### Product Review

A product review is a customer review of a product or service made by a customer (Bueno et al., 2019). Amazon and Alibaba are two e-commerce platforms that allow members of the community to review and evaluate their products. Consumers, marketing departments, and shopping platforms can all benefit from product reviews posted online (Kwak et al., 2019). Hence, product review is the important factor that influences consumer tendency in buying commodities in E-commerce.

**H3:** *Product review influences consumer tendency in buying commodities in E-commerce*.

### Product Quality

Product quality encompasses all of a product's features and abilities to meet consumer expectations and provide satisfaction (Susanti and Jasmani, 2020). If the quality of the product is poor, then people will not trust or purchase the product. Product quality has a greater impact on the consumer tendency of buying commodities (Liu et al., 2022). Based on the above-mentioned concepts, we included the hypothesis that the quality of a product influences consumer tendency.

**H4:** *Product quality influences the consumer tendency in buying commodities in e-commerce*.

### Promotion

Promotion is a strategy of communication between buyers and sellers, which helps the companies to introduce their commodities to the consumers (Wall, 2022). The goal of commodity promotions is to influence consumer tendency of buying by presenting a new commodity that will be aimed at introducing it to new customers (Luo et al., 2021). Based on the above-mentioned concepts, we included the hypothesis that the promotion of a commodity influences consumer tendency.

**H5:** *Promotion influences customer tendency in buying commodities in e-commerce*.

## Methodology

### Sample and Procedure

This study's sample size was calculated using a basic rule of thumb for successful PLS-SEM estimation. The minimal sample number is 10 times the greatest number of pathways, aiming at any construct in the outer model, according to a popular rule of thumb (Hahm et al., 2022). Product quality, which contains 16 questions, is the variable with the most questions in this study. As a result, the minimal number of samples in this investigation should be 160, according to the established rule of thumb for robust PLS-SEM estimations. As a result, online questionnaires were given to China consumers who follow, like, or comment on social media pages to gather information. After removing invalid questions, a total of 385 surveys from China consumers were obtained.

### Research Findings and Discussion

#### Scale Validity and Reliability

The Table 2 shows that between 0.603 and 0.803, Cronbach's alpha is bigger than.6. Between 0.794 and 0.872, compound reliability (CR) is better than 0.7. Between 0.561 and 0.649, the average variance extraction (AVE) is >0.5. As a result, it meets the convergence validity criterion of the measurement model using PLS-SEM, indicating that the measuring scale used in this work is convergent.

**Table 2:** Validity and reliability for constructs.

**Constructs**	**Cronbach's alpha**	**CR**	**AVE**
Price	0.720	0.841	0.644
Brand loyalty	0.733	0.846	0.649
Product Review	0.803	0.872	0.627
Product quality	0.742	0.837	0.565
Promotion	0.603	0.794	0.561

#### Structural Equation Model (SEM)

The findings of a hypothesis test are revealed using the structural equational model (SEM) analysis. Table 3 shows that product price significantly influences consumer tendency (β = 0.194; *p* < 0.001), brand loyalty significantly influences consumer tendency (β = 0.158; *p* < 0.001), product review significantly influences consumer tendency (β = 0.178; *p* < 0.001), product quality significantly influences consumer tendency (β = 0.165: *p* < 0.001) and (β = 0.258; *p* < 0.001) product promotion significantly influences consumer tendency of commodities in E-commerce. As a result, all of the hypotheses were supported: Hypothesis 1, Hypothesis 2, Hypothesis 3, Hypothesis 4, and Hypothesis 5.

**Table 3:** Structural equation model analysis.

**Hypotheses**	**Relationship**	**Beta**	***P* Values**	**Decision**
Price	Price consumer tendency	0.194	0.000	Supported
Brand loyalty	Brand Loyalty-consumer tendency	0.158	0.000	Supported
Product review	Product review-consumer tendency	0.178	0.000	Supported
Product quality	Product quality-consumer tendency	0.165	0.000	Supported
Promotion	Promotion-consumer tendency	0.258	0,000	Supported

## Conclusion

With the increasing rise of e-commerce sites in modern days, perceptual analysis of product evaluations technology has gotten a lot of attention. The basic rule for effective PLS-SEM evaluation was used to compute the sample size for this study. The elements of consumer sentiment tendency are examined in detail in this review study. First, pricing influences buying decisions, particularly for commonly purchased items, and decides which retailer, product, and brand will be preferred. When it comes to selecting what benefits they wish to obtain from acquiring goods or services, consumers are more cautious. Brand loyalty was more profitable than it was discussed in this study. Third, Amazon and Alibaba are two e-commerce sites that allow community members of the public to rate and review products. Fourth, commodity advertising aims to influence consumer purchasing decisions by promoting a new product. And, last, the quality of a product has a stronger influence on a consumer's desire to purchase goods. Only five elements of consumer sentiment tendency were identified in this study. More can be added in the future for further research.

## Data Availability Statement

The original contributions presented in the study are included in the article/supplementary material, further inquiries can be directed to the corresponding author.

## Ethics Statement

Ethical review and approval was not required for the study on human participants in accordance with the local legislation and institutional requirements. Written informed consent from the patients/participants legal guardian/next of kin was not required to participate in this study in accordance with the national legislation and the institutional requirements.

## Author Contributions

The author confirms being the sole contributor of this work and has approved it for publication.

## Conflict of Interest

The author declares that the research was conducted in the absence of any commercial or financial relationships that could be construed as a potential conflict of interest.

## Publisher's Note

All claims expressed in this article are solely those of the authors and do not necessarily represent those of their affiliated organizations, or those of the publisher, the editors and the reviewers. Any product that may be evaluated in this article, or claim that may be made by its manufacturer, is not guaranteed or endorsed by the publisher.
